# A General Method to Discover Epitopes from Sera

**DOI:** 10.1371/journal.pone.0157462

**Published:** 2016-06-14

**Authors:** Kurt Whittemore, Stephen Albert Johnston, Kathryn Sykes, Luhui Shen

**Affiliations:** Center for Innovations in Medicine, Biodesign Institute, Arizona State University, 1001 South McAllister Avenue, Tempe, Arizona 85287, United States of America; New York State Dept. Health, UNITED STATES

## Abstract

Antigen-antibody complexes are central players in an effective immune response. However, finding those interactions relevant to a particular disease state can be arduous. Nonetheless many paths to discovery have been explored since deciphering these interactions can greatly facilitate the development of new diagnostics, therapeutics, and vaccines. *In silico* B cell epitope mapping approaches have been widely pursued, though success has not been consistent. Antibody mixtures in immune sera have been used as handles for biologically relevant antigens, but these and other experimental approaches have proven resource intensive and time consuming. In addition, these methods are often tailored to individual diseases or a specific proteome, rather than providing a universal platform. Most of these methods are not able to identify the specific antibody’s epitopes from unknown antigens, such as un-annotated neo antigens in cancer. Alternatively, a peptide library comprised of sequences unrestricted by naturally-found protein space provides for a universal search for mimotopes of an antibody’s epitope. Here we present the utility of such a non-natural random sequence library of 10,000 peptides physically addressed on a microarray for mimotope discovery without sequence information of the specific antigen. The peptide arrays were probed with serum from an antigen-immunized rabbit, or alternatively probed with serum pre-absorbed with the same immunizing antigen. With this positive and negative screening scheme, we identified the library-peptides as the mimotopes of the antigen. The unique library peptides were successfully used to isolate antigen-specific antibodies from complete immune serum. Sequence analysis of these peptides revealed the epitopes in the immunized antigen. We present this method as an inexpensive, efficient method for identifying mimotopes of any antibody’s targets. These mimotopes should be useful in defining both components of the antigen-antibody complex.

## Introduction

Characterizing the interactions between disease-specific antibodies and their cognate antigens has proven highly informative in the study of host-pathogen relationships and critical in the development of effective biomedical products. The discovery of modified antigens or autoantigens that are specifically recognized by patient antibodies is of growing importance in disease research and target development for diagnostics, vaccines, and therapeutics. These complexes are typically found by querying immune sera against possible ligands in lysates, or in libraries of proteins or peptides made recombinantly or synthetically. Myriad of binding assays such as immunoblots [1], ELISAs [2], phage display [3], ribosome display [4], beads [5], and microarrays [6, 7] have been employed to identify the antigen or epitope recognized by an antibody. Here we explore using a simple, universal system for epitope identification.

In its original description, phage display was used to survey a library of peptides for binding to a given antibody[8]. It has now been used extensively to display libraries of peptides or antibody fragments, expressed as coat protein fusions on the phages surface, for “panning” against a particular molecule of interest. Phages are washed across an isolated, immobilized target; bound recombinant phage are collected and amplified in bacteria for additional rounds of panning [9]. One of the major drawbacks of phage display is the technique’s reliance on multiple in vivo steps that cannot be well-controlled and incur biases to the output. For example, any peptide-coat protein fusions that reduce the fitness of the phage or reduce secretion to the phage surface will not be well represented if at all. In the initial panning round each phage recombinant is present in such limited numbers that the probability of a ligand finding a target can be stochastic. Yet only those recombinants that survive this first round are subsequently propagated reiteratively. Phage approaches do not lend themselves to high throughput of samples. As an alternative, in vitro translation systems such as ribosome display have been developed for studying protein-protein interactions, including antigen/antibody binding. Like phage, very large libraries can be constructed at minimal cost but the diversities of these recombinant mixtures are difficult to maintain and are not reproducible. In addition, apparent diversities can be misleading since the redundancy of the genetic code, incidental stop codons, and peptide-dependent effects on translation efficiency will limit the ultimately displayed diversity.

In vitro combinatorial synthesis of peptides on beads and microarrays of either proteins or peptides have been explored as library formats for surveying target binding [5, 10]. Both of these methods are performed entirely in vitro, and thereby resolve the vagaries of in vivo propagation and biological compatibility. Since peptides are used directly, the issues of translating DNA are avoided. For the libraries in bead format, the binding steps must be followed by decoding what is bound through peptide-sequencing, chemical-tracking, or other reading methods. The synthesis, binding, and decoding steps tend to be laborious, time consuming, and often lack reproducibility [11]. Array based libraries are more efficient and reliable.

However most of the applications of the above methods focused on known biological targets. This limited their applications. Each high throughput library can only cover limited proteome. On the other side, the antigens or epitopes within the antigen may not be in the known proteome. Studies can be confounded by the fact that immune sera often carry antibodies to mutant, unknown, or even exogenously-derived antigens of a host or pathogen proteome.

Random peptide libraries, whether in vivo or in vitro, provide a means for identifying mimotopes of unknown antigens and are far more economical since one library can be used for all screens. For example, an antibody panned against phages displaying random sequence peptide fusions might select recombinants that mimic or hold similarity to a previously unknown ligand [12]. Microarrays consisting of random sequence peptides or peptoids have been explored for identifying ligands [13]. Probing of a SPOT synthesis array carrying 5520 random 15-mers with three different monoclonal antibodies followed by substitutional analysis was able to identify mimotopes of the known wild type epitopes [14]. However, the application of the random peptide array was limited by the libraries size and the cost and was only applied to monoclonal antibodies.

Recently, the immunosignatures technology has been developed as a universal platform to profile complex antibody mixtures [15]. This technology is based on using arrays of peptides of 10 to 17 amino acids in length chosen form non-natural sequence space. To maximally represent sequence diversity, which excludes the peptides sequences that are exactly matched to a natural peptide in the NCBI reference protein database. The random peptide library can be expanded up to 330,000 unique sequences [16]. The peptide can be printed from pre-synthesized stocks or in situ synthesized. Such a library of random sequence peptides can be used to identify the disease specific antibody profile from patient sera, including the infection diseases and different cancers [17–19] and predict vaccine efficacy with the specific antibody profile as well[20]. Based on the sequence analysis of the array peptides that were specifically recognized by the antibody or serum from infectious diseases, we can identify the epitopes to the antibodies and track the sequences back to the known antigens that relate to the disease [20–22]. However, there were two limitations. One is that an antibody could be to an unannotated sequence. The other is that the actual antibody to the epitope was not created as a reagent.

Here we employ the IMS as a universal platform to design a method for identifying mimics of disease-relevant epitopes and then show that these mimotopes can capture the disease-relevant antibodies for the further investigation, such as identification of the unknown biological antigens.

Unique from other platforms that focused on analyzing the purified targets [6, 7] the methods we demonstrate directly use the serum for the efficient screen for mimics of disease-relevant epitopes. The target epitope may not be highly immunogenic and the antibodies recognizing the target epitope in the serum may be mixed with irrelevant antibodies with high affinity to other antigens. Based on these complex conditions, we designed an experiment to identify mimotopes of a tumor-associated mutant, SMCfs, from a complex immune serum that contains high affinity polyclonal antibody to the KLH protein. SMCfs is a 17 amino acid novel, tumor specific frame-shift peptide derived from the mis-splicing RNA of the structural maintenance chromosome 1A protein (SMC1A). The transcript of the SMCfs peptide was first found in a human EST database. The non-natural sequence peptide microarray, CIM10K, contains 10,000 non-natural sequence peptides chosen from random sequence space. We have shown that the random sequence peptide microarray can identify the epitopes that are recognized by monoclonal antibodies [21, 22]. We demonstrate here that this non-natural sequence peptide microarray can also be used to identify specific mimotopes for antibody enrichment from a complex immune serum sample. Sequence analysis can identify relevant epitopes of the antibody responses.

## Material and Methods

### Peptides and beads

A number of free peptides were synthesized by Sigma, Inc. Beads for antibody affinity purification were prepared in the lab. Peptides were directly synthesized on the TentaGel Beads. The sequences of these peptides and their assigned names are presented in Table 1.

**Table 1:** Amino acid sequences of free peptides and peptide-bead conjugates.

Assigned Name	Sequence	Description
SMCfs	CCGIYCHEEPQREDSSI	human SMC1A frameshift 17mer peptide
SMCfs-27	TAIIGPNGSGCCGIYCHEEPQREDSSI	human SMC1A frameshift 27mer peptide
RP1	TISKYVMVEPMRQHEEWGSC	random peptide SMCfs mimotope
RP2	AVSHQEMNEGEQGPMREGSC	random peptide SMCfs mimotope
RP3	RVGEMPMREYDISGGSGGSC	random peptide SMCfs mimotope
RP4	TAFYRTLTKHEVDPGIAGSC	random peptide SMCfs mimotope
CP1	AVLLMCQLYQPWMCKEYYRLL	negative control peptide which is not a mimotope of SMCfs
SMCfs-B	CCGIYCHEEPQREDSSI	human SMC1A frameshift 17mer peptide conjugated to Tentagel beads
RP1-B	TISKYVMVEPMRQHEEWGSC	random peptide SMCfs mimotope conjugated to Tentagel beads
RP2-B	AVSHQEMNEGEQGPMREGSC	random peptide SMCfs mimotope conjugated to Tentagel beads
RP3-B	RVGEMPMREYDISGGSGGSC	random peptide SMCfs mimotope conjugated to Tentagel beads
RP4-B	TAFYRTLTKHEVDPGIAGSC	random peptide SMCfs mimotope conjugated to Tentagel beads
CP2-B	ATKAAIPGPNTVPRAP	negative control peptide which is not a mimotope of SMCfs conjugated to Tentagel beads

### Rabbit anti-SMCfs sera

Anti-human SMC1Afs serum was generated by Global Peptide Service LLC (Fort Collins, CO). The 17 amino acid (a.a.) SMC1A frame shift mutant sequence (SMCfs), identified in human tumor cDNA (CCGIYCHEEPQREDSSI), was synthesized by the peptide synthesis lab at Arizona State University and then conjugated to keyhole limpet hemocyanin (KLH) by Global Peptide Service LLC.A New Zealand white rabbit was immunized with the SMCfs-KLH conjugate. Blood for the experiment was collected at exsanguinations after two immunizations.

### ELISA

ELISA plates were coated with 50 μL of 10 μg/mL of peptide or protein in carbonate coating buffer and incubated at 4°C overnight. The coated plates were washed 3X with PBST and blocked with 200 μL of 3% BSA in PBST at 37°C for 30 minutes. The blocked plate was washed 3X with PBST and 50 μL of primary anti-serum or purified antibody diluted in 3% BSA in PBST was applied. The plate was then incubated at 37°C for 1 hr. After the incubation, the plate was washed 3X with PBST. The antibody was detected with 50 μL HRP-goat anti-rabbit IgG diluted 1:2000 in 3% BSA in PBST. After the plate was incubated at 37°C for 1 hr, the plate was washed 3X and developed with 50 μL TMB for 10 minutes at room temperature. The development was stopped by adding 50 μL of 0.5 N HCl, and the plate was read with a SpectraMax 190 Molecular Devices instrument at OD 450 nm.

### Antibody absorption

Specific antibodies were absorbed from the rabbit anti-SMCfs sera by applying the sera to the SMCfs coated plate. The rabbit sera was diluted 1:250 with 3% BSA in PBST and incubated with the SMCfs peptide coated plate at 37°C for 1 hr. The unbound antibody in the supernatant was then removed and applied to another peptide coated well to remove more antibody specific for the peptide. This process was repeated up to 20 times, and this serum was then later applied to the peptide microarray at a dilution of 1:500. This same method was used to produce negative control antibody absorbed sera using the negative control CP1 peptide.

### Non-natural sequence peptide array (CIM10K array) printing

The 10,000 non-natural randomly generated peptide sequences were custom synthesized by Sigma, Inc. These 20-mers were designed with 17 non-natural sequence a.a. residues (excluding cysteine) and a 3 amino acid (GSC) linker sequence on the C terminus. The C terminal cysteine binds to a sulfo-SMCC coated aminosilane glass slide. The solutions of different non-natural sequence peptides were printed onto the glass slide using a Nanoprint 60 instrument (Array It Technologies).

### Application of sera to non-natural sequence peptide array

Rabbit sera samples were applied to the non-natural sequence peptide microarray using a Tecan HS 4800 Pro microarray hybridization station. Slides were first washed for 30s with TBST, and then blocked with a blocking buffer consisting of BSA, mercaptohexanol, Tween 20, and PBS for 1 hr at 23°C. Duplicate samples of sera were diluted 1:500 in an incubation buffer consisting of BSA, Tween 20, and PBS and incubated with the slide for 1 hr at 37°C. The slide was then washed, and 5 nM of goat anti-rabbit IgG conjugated with Alexa Fluor 647 dye was applied for 1 hour at 37°C. The slide was then washed and dried for 5 min.

### Scanning and analysis of array

The slides were scanned with an Agilent Technologies DNA Microarray Scanner with Surescan High-Resolution Technology instrument and analyzed with GenePix Pro 6.0 software to determine the fluorescence intensity of each spot. GeneSpring GX 7.3, Microsoft Excel, simple custom Java code, and GraphPad Prism 4 were then used to perform further analysis of this data.

### Antibody affinity purification

Total IgG of the rabbit anti-SMCfs sera was purified using Pierce Protein A/G Agarose beads according to the protocol of the manufacturer. 1 ml of the TentaGel beads with specific peptides were embedded in a column. 3 ml of the purified total IgG was added to the column and incubated with the beads for 1 hr at room temperature. The column was then washed with 10 ml PBST. The specific IgG was eluted with 4 fractions of 1 ml IgG Elution Buffer (Pierce, Inc) and each fraction was neutralized with 100 ul 1M TRIS (pH8.0). All of the eluted fractions were measured at an absorbance of 280nm. The two fractions with the highest absorbance were combined and used for further analysis at a 1 to 40 dilution in 3% BSA in PBST.

### Analysis of motifs

GLAM2 software [23] was used to identify and score common motifs among the high binding peptides. Bepipred was used to find and score B cell epitopes [24].

## Results and Discussion

### Screening of anti-SMCfs serum on non-natural sequence peptide array

**Fig 1:**
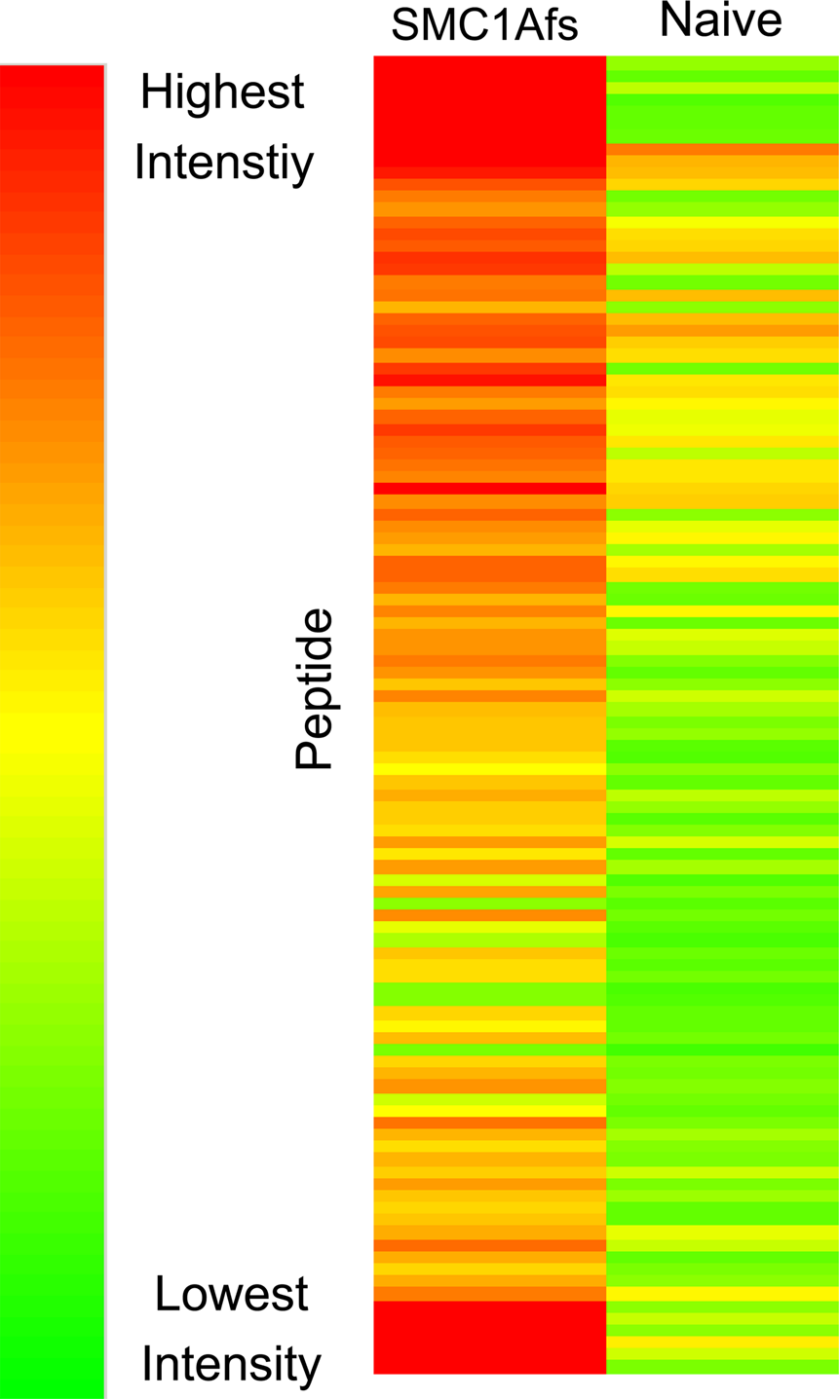
Selective binding of anti-SMCfs serum to a library of non-natural sequence peptides printed on a microarray. Polyclonal rabbit serum generated against the SMCfs peptide conjugated to KLH and naive rabbit serum were applied to an array of 10,000 synthetic peptides of randomly generated sequence. Student’s t-test analyses comparing the probing results identified 108 non-natural sequence 20-mers with differential binding to the immune versus naïve sera (p-values <0.0001). These differences in peptide-binding intensities are visually represented as a heatmap.

To identify non-natural sequences recognized by the immune serum, both naïve and SMCfs-immune rabbit sera were applied to the array. We identified a total of 108 non-natural sequence peptides which exhibited high specificity to the immune serum relative to the naïve rabbit control serum based on the t-test (p<0.0001)(Fig 1, S1 Table). The anti-SMCfs immune serum contains polyclonal antibodies to both SMCfs peptide and KLH, the carrier protein. Therefore, these 108 peptides carry potential mimotopes of either the SMCfs peptide or KLH protein.

### Sequence analysis to identify the mimic epitope to KLH protein

Since the KLH is a large and highly immunogenic antigen, the rabbit anti-serum contained high titer antibodies to the KLH protein. Mimotopes to KLH among the 108 SMCfs peptides were investigated (S2 Table). When the 108 peptides selected for higher binding to SMCfs sera than to naive sera are compared to the KLH1 and KLH2 sequences (a total of 6,830 aa) with GLAM2 individually, then 54 of the 108 peptides have a GLAM2 score above 11.7. The total collection of scores ranges from 7.93 to 17.8 with 11.7 at the 50th percentile. Among the top 54 peptides by GLAM2 score, 2 contain mimotopes that align with HAEDHFYI, 2 with HHEKHHEDHH, 2 with DSFDYQNRFRY, 2 with DKEYYDVWRWRNKVMPNPFA, 2 with HHANTDRIW, 2 with WGFYRAYHF, and the rest of the mimotopes align with various regions of the KLH protein sequence. These results demonstrate again that the IMS could efficiently identify the epitopes to the known antigens directly from the complex serum.

### SMCfs immune serum absorption and array analysis

**Fig 2:**
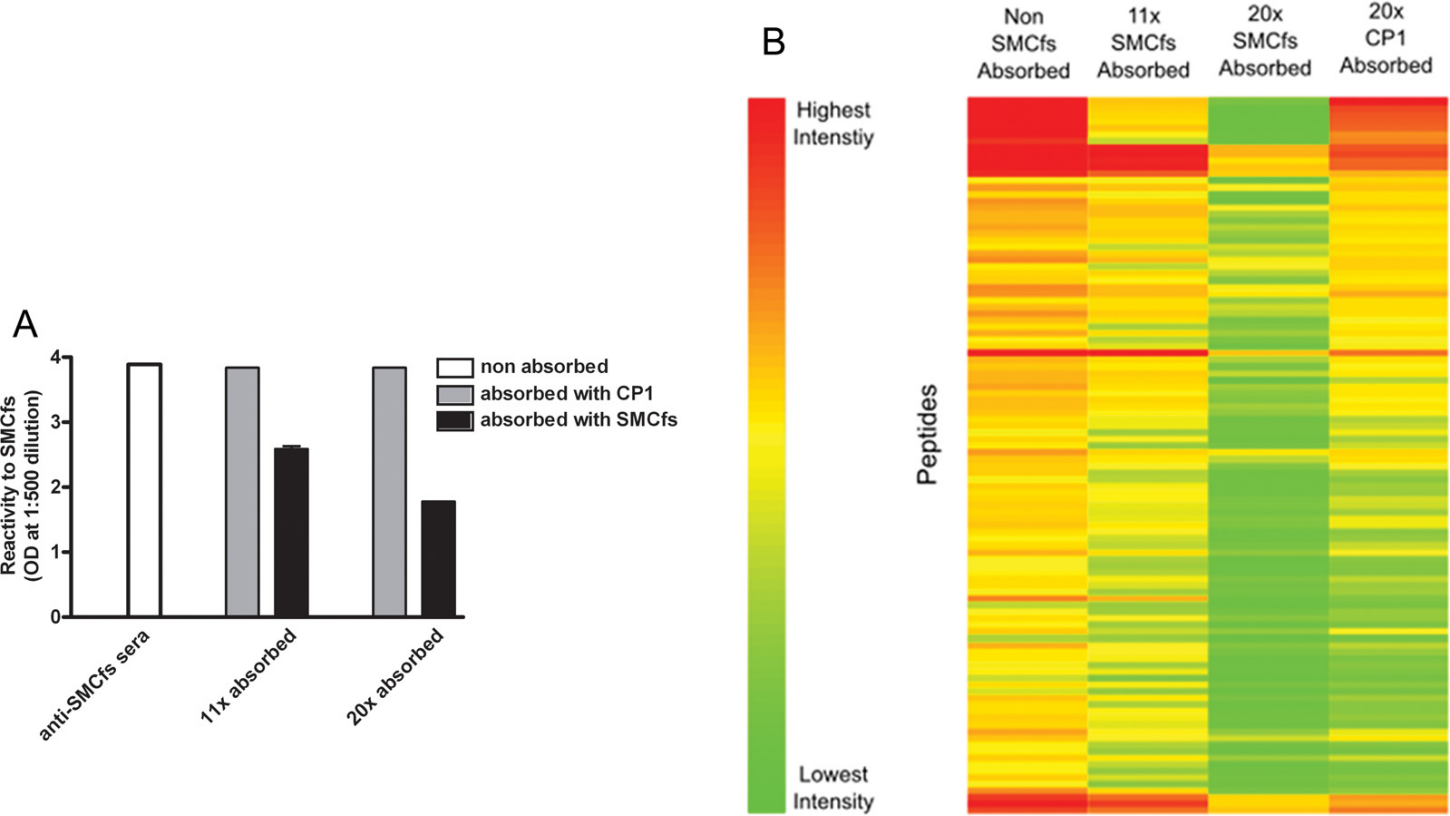
Analyses of anti-SMCfs serum pre-absorbed against SMCfs peptide. **A. Validation of SMCfs antibody depletion from immune serum.** The serum-absorption steps (11or 20) refer to the number of iterative rounds of SMCfs-peptide or CP1-peptide absorption experiments conducted before application of the depleted serum to ELISA plates coated with SMCfs peptide. Error bar indicates SD of the duplication. **B. Signal intensity changes in anti-SMCfs serum binding following peptide-specific depletion.** The depleted immune sera samples were applied to the peptide array. Binding intensities to the 108 non-natural sequence peptides, which were shown to be selectively recognized by the original anti-SMCfs serum, are displayed as a heatmap for their visual comparison. There is an 850 fold difference between the lowest fluorescence intensity and the highest intensity. Four anti-SMCfs sera samples were applied to the peptide array, 1) non-absorbed anti-SMCfs serum, 2) anti-SMCfs serum absorbed 11x against SMCfs peptide, 3) anti-SMCfs serum absorbed 20x against SMCfs peptide, 4) anti-SMCfs serum absorbed 20x against the CP1 negative control peptide.

Compared to KLH, the SMCfs peptide is much smaller and lower immunogenic. To distinguish the mimotopes of the SMCfs peptide from that of KLH, we specifically depleted the anti-SMCfs antibodies from the immune serum through iterative antibody absorption steps. ELISA analysis confirmed that anti-SMCfs antibodies were partially depleted from the original serum after 11 rounds of absorption and further depleted after 20 rounds (Fig 2A).

The anti-SMCfs antibody-depleted samples, as well as the negative control serum, were applied to the CIM10K array. Reactivity profiles of these samples against the 108 peptides selected in the original screen were compared (Fig 2B, S3 Table). Different binding profiles were observed among the differentially depleted anti-SMCfs serum samples. Peptide mimotopes of SMCfs would be predicted to react similarly in the presence of the non-absorbed and control-peptide absorbed (anti CP1-antibody depleted) immune serum samples. On the other hand, peptides carrying a SMCfs mimotope would be predicted to display decreased reactivity in the presence of the anti-SMCfs antibody depleted serum sample. Based on these criteria of specificity, we developed a formula to score each of the 108 peptides (Eq 1). Four top scoring peptides, designated RP1, RP2, RP3, and RP4, were re-synthesized for testing as functional mimotopes.

#### Eq 1. Score assignment for random peptide SMCfs specificity

A high score indicates that the given random peptide binds to antibodies with high specificity for the SMCfs sequence, and a low score indicates the opposite. S_max_ is the maximum signal intensity of the peptide with unabsorbed SMCfs sera. S_20X_ is the intensity of the peptide with sera after SMCfs binding antibodies have been absorbed twenty times. S_CP_ is the intensity of the peptide with sera after CP1 binding antibodies have been absorbed twenty times. The CP1 peptide is a negative control peptide with no sequence similarity to SMCfs.

$$\frac{S_{max}-S_{20X}}{S_{max}}+\frac{S_{CP}}{S_{max}}$$ (1)

### ELISA validation of the binding specificity of RP1-RP4 to anti SMCfs immune serum

**Fig 3:**
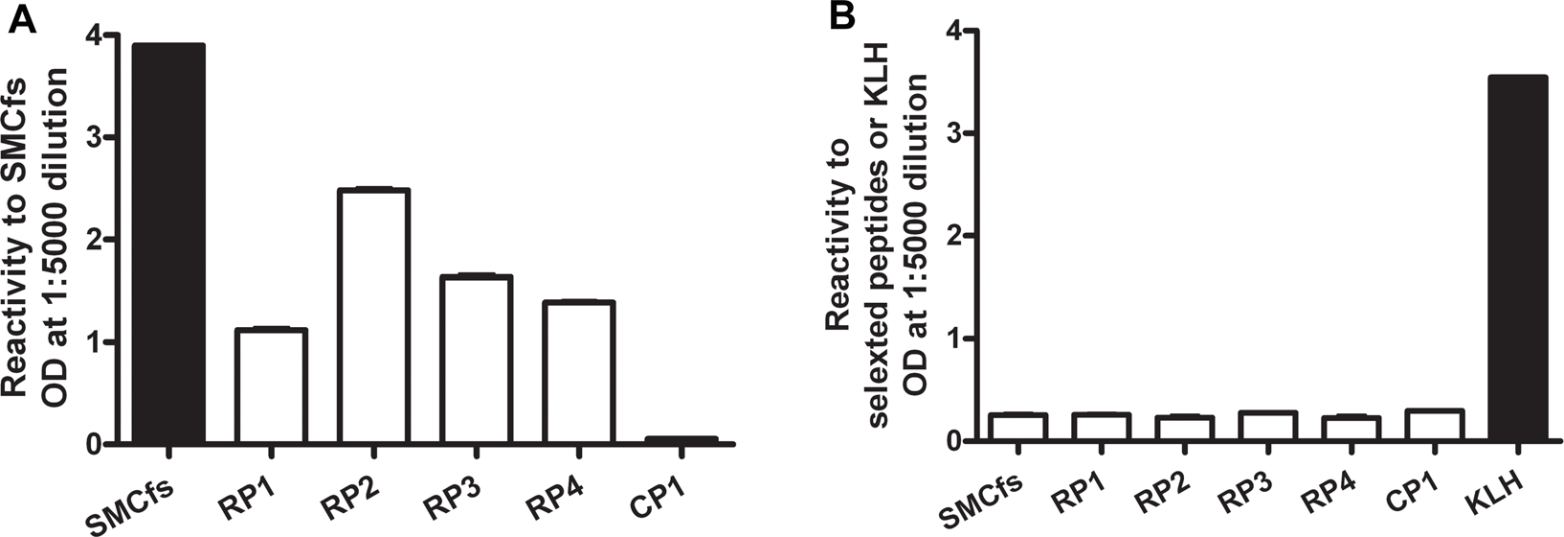
ELISA determinations of anti-SMCfs sera binding to array-selected peptides. **A. Immune serum.** Rabbit polyclonal anti-SMCfs serum was applied to the SMCfs peptide, four of the array-selected peptides, and a control peptide. The SMCfs peptide is the positive control. **B. Control serum.** Mouse polyclonal anti-KLH serum was applied to the same set of peptides described in A. The KLH is the positive control. Error bar indicates SD of the duplication.

To assess the specificity of anti-serum binding to the four putative mimotopes, we first analyzed the reactivity of the selected peptides to the immune serum by ELISA (Fig 3A). Compared to the negative control peptide CP1, all four mimotopes had significant though varied reactivity levels to the immune serum. However, the reactivity levels of the four mimotopes were lower than that of the original SMCfs peptide, which had a saturated signal at 1:5000 dilution.

To test the possibility that RP1-RP4 are mimotopes of a KLH protein epitope rather than SMCfs, we measured the reactivity of the four selected random peptides, the original SMCfs peptide, and CP1 to the polyclonal KLH immune serum (Fig 3B). All six of these peptides showed similarly low reactivity levels when probed with the KLH immune serum, whereas the KLH protein-antigen displayed saturated signal against the anti-KLH serum. These ELISA analyses indicate that the four random peptides act as mimotopes of the SMCfs peptide; and indicate that each mimotope contains a partial or suboptimal epitope of the cognate SMCfs peptide.

### Affinity purification of anti-SMCfs antibodies with the array-selected peptides

**Fig 4:**
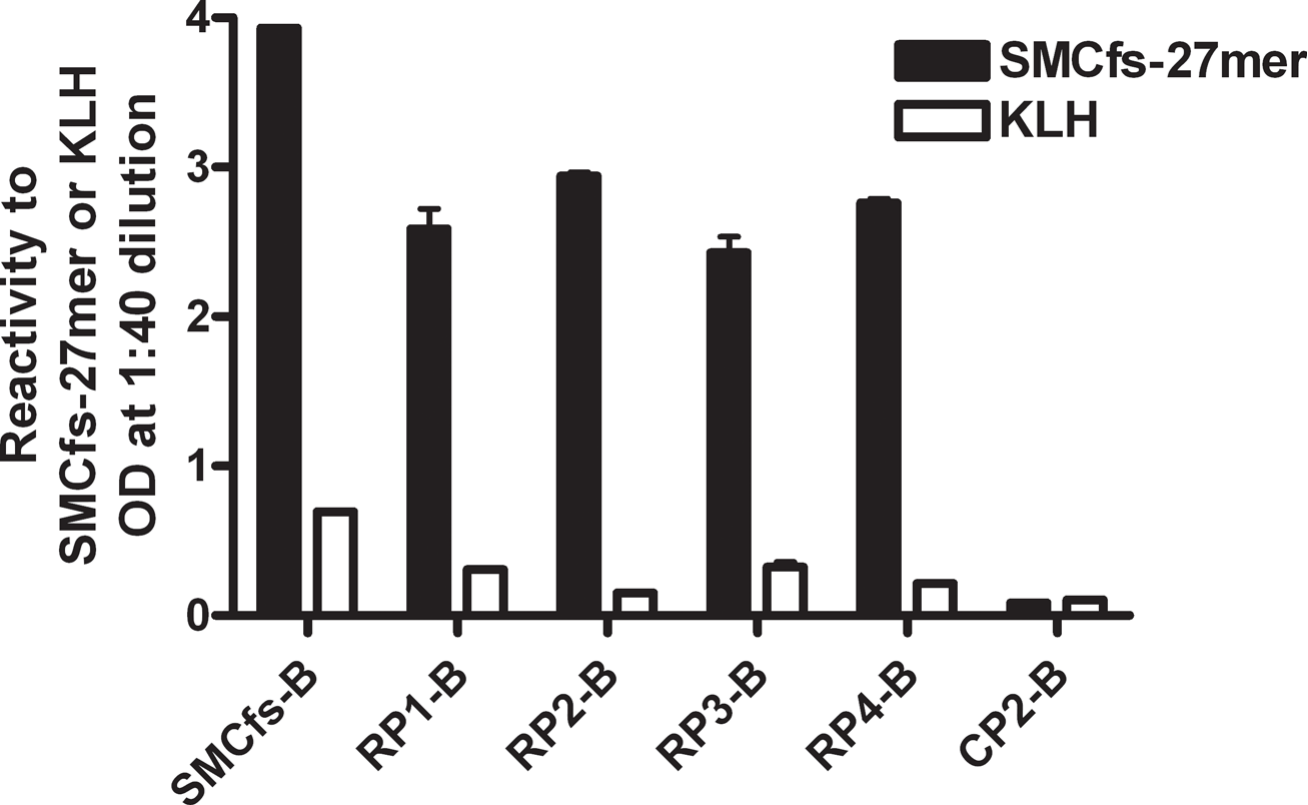
ELISA analysis of affinity-purified antibodies. The SMCfs peptide, four highly array-reactive peptides, and a control peptide (CP2-B) were synthesized on Tenta-gel beads. These were used to prepare individual affinity columns. Purified total IgG of the anti-SMCfs serum was applied; bound antibodies were eluted. These peptide-purified antibody samples were analyzed by ELISA against SMCfs-27 coated or KLH coated plates. Error bar indicates SD of the duplication.

To determine whether RP1 to RP4 can be used to selectively purify antibodies specific to the original immunogen, we prepared affinity purification columns with each of four random peptides (RP1 to RP4), SMCfs, and an irrelevant peptide as negative control (CP2-B). The binding specificities of the affinity-purified antibodies were measured by determining the activity of the eluted antibodies against SMCfs-27 or KLH antigen (Fig 4). The SMCfs-27 is comprised of the sequence of SMCfs plus the 10 amino acids found in the wild type protein immediately upstream of the frame-shift. The SMCfs-27and SMCfs peptide had similar reactivity to the SMCfs immune serum (data not shown). The antibodies purified by RP1-RP4 specifically recognized the original immunogen, not the KLH protein. This result confirms the specificity of the mimotopes screened by the CIM10K array, and validates this approach of using a mimotope to purify specific antibodies from a complex antibody mixture.

### Measuring cross-reactivity of anti-SMCfs antibodies to antigen mimotopes

**Fig 5:**
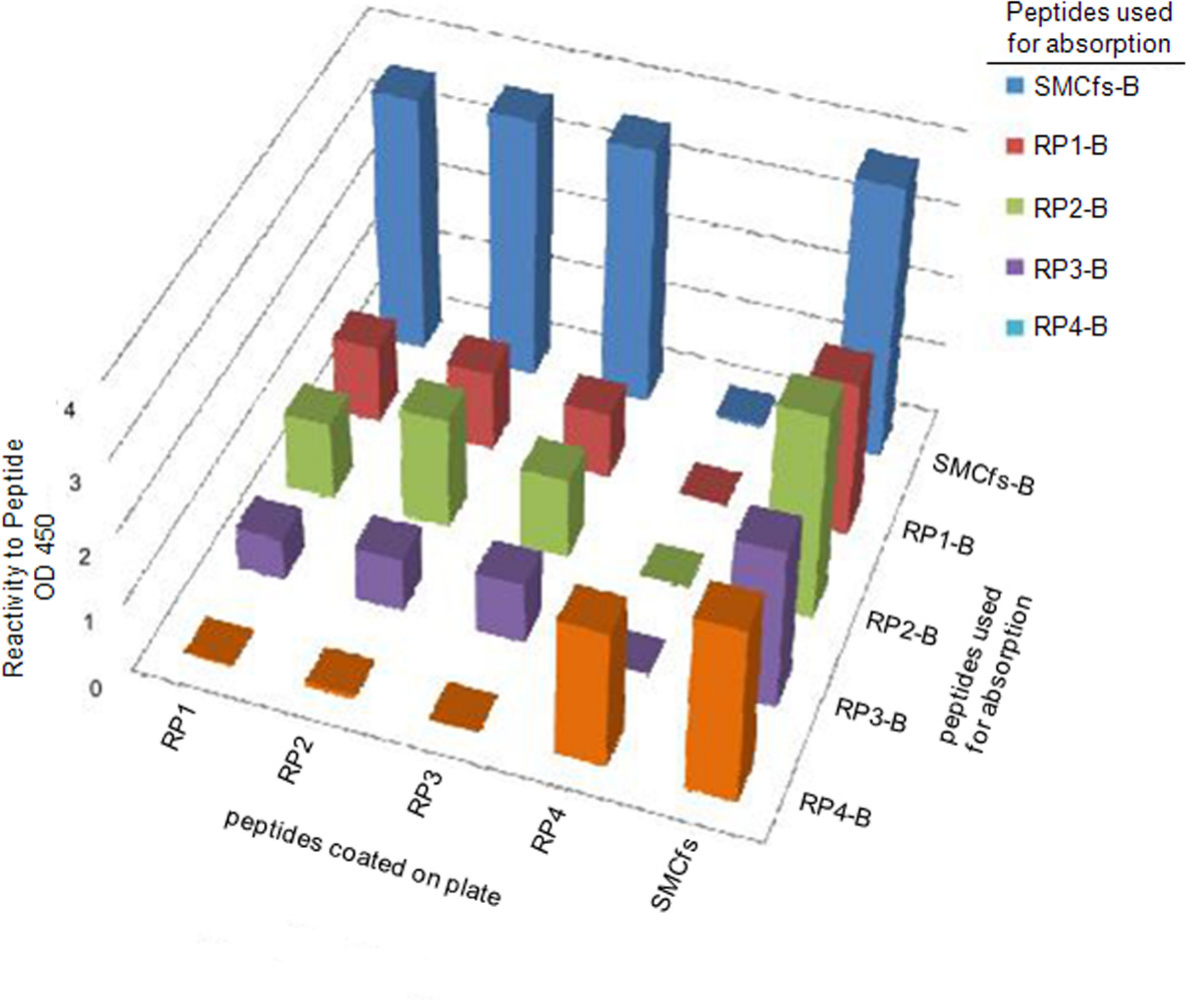
Differential binding of affinity-purified anti-SMCfs antibodies to SMCfs versus library-derived and mimotopes. The antibodies affinity purified by SMCfs-B or four library-selected peptide bound to beads (RP1-B through RP4-B) were measured for reactivity against the SMCfs and mimotope peptides by ELISA and displayed in a three-dimensional bar graph.

The antibody samples that were affinity-purified from the SMCfs immune serum with the SMCfs peptide or with the array-selected mimotopes display more than one cross-reactivity group as measured by ELISA (Fig 5). Antibodies purified with the RP1, RP2, or RP3 peptides cross-reacted strongly with one another; however, antibodies purified with the RP4 only recognized the RP4 peptides. One explanation for this behavior is that RP1, RP2, andRP3 possess a similar sequence or structural motif whereas RP4 carries a unique one. Sequence analysis described in section 3.6 below supports this hypothesis. Notably, all four mimotope-purified antibody samples display the highest reactivity levels against the cognate SMCfs immunogen, rather than the sequence ligand used to purify them. This suggests three points. First, the non-natural sequences appear to have been selected from the arrayed-library based on their SMCfs-like binding characteristics, as opposed to binding to some perhaps related but different target. Second, the sampling of non-natural sequence space afforded by this library may be too sparse to provide mimotopes that are close enough to the true SMCfs epitopes to achieve higher binding activity. Notably, the CIM10K array displays 10^4^ different peptide sequences; whereas 10^21^ unique 17-mers are possible. Third, even for a peptide as short as 17aa, the mimotopes can resolve two different binding sites.

### Sequence Analyses to identify the potential epitope in the unknown antigen

From the mimotpe identification to the validation, we did not use the sequence information of the original SMCfs peptide. To test the possibility of using the sequence analysis of the mimotopes to identify epitope of the unknown antigen, we did the sequence analysis of both the SMCfs and RP1 to RP4 peptides. The epitope search software Bepipred was used to predict strong B cell epitopes within the unique SMC1A frameshifted sequence. The highest amino acid score was assigned to the proline within HEEPQRE. The GLAM2 software was used to identify sequences in RP1 through RP4 with similarity to sequence stretches found in SMCfs. The RP1 peptide contains two motifs: HEE and YXXXXPMRQ, although they are in reverse order relative to those sequences of SMCfs. Note that previous experiments have also identified high binding peptides with inverted sequences relative to the original epitope, but these inversions typically involved just two amino acids [25]. RP2 contains PMREGS and RP3 contains EMPMRE. RP4 contains only the simple dimer HE (Table 2). This analysis shows that three of the four peptides contain a PQRE-like motif found in the cognate SMCfs peptide. Although RP1 contains reversed YXXXXPMRQ and HEE motifs, the peptide is specifically recognized by anti-SMCfs antibodies. This may indicate that i) the HEE motif acts independently of the PQRE-motif, ii) they work in concert but order is not important, iii) or that HEE is not relevant to binding. There is no conserved linear motif of the SMCfs in the RP4 suggesting that RP4 may contain the mimotope with the similar topological structure. This will be another advantage of the random peptide library for the mimotope screen.

**Table 2:** Motifs in common between RP1-4 and SMCfs as identified by the GLAM2 software.

Sequence ID	Sequence	Motif in Peptide Matching with SMCfs	Matching SMCfs sequence
SMCfs	CCGIYCHEEPQREDSSI	-	-
RP1	TISKYVMVEPMRQHEEWGSC	YXXXXPMRQ	YXXXXPQRE
		PMRQ	PQRE
		HEE	HEE
RP2	AVSHQEMNEGEQGPMREGSC	PMREGS	PQREDS
		PMRE	PQRE
RP3	RVGEMPMREYDISGGSGGSC	EMPMRE	EEPQRE
		PMRE	PQRE
RP4	TAFYRTLTKHEVDPGIAGSC	HE	HE

Note that the RP1-4 peptides which have been the focus of this paper were not the only peptides among the 108 selected peptides which exhibited good mimotope matches of the SMCfs peptide (S4 Table). When the 108 peptides selected for higher binding to SMCfs sera than to naive sera are compared to the SMCfs peptide with GLAM2 individually, then 56 of the 108 peptides have a GLAM2 score above 4.50. The total collection of scores ranges from 2.31 to 8.70 with 4.55 at the 50th percentile. The 5 high intensity peptides have GLAM2 scores that place them in the top 22 peptides of the 108 peptide list sorted by GLAM2 score. The top 4 peptides ranked by SMCfs score are in the top 16 peptides of the 108 peptide list sorted by GLAM2 score. Therefore, there seems to be a good correspondence between intensity detected from the array and the GLAM2 score for each peptide. Among the top 56 peptides by GLAM2 score, 28 contain mimotopes that align with GIYCH, 30 contain mimotopes that align with CHEEPQRE, and 6 contain mimotopes that align with DSSI of the CCGIYCHEEPQREDSSI SMCfs peptide.

There was a good correspondence between GLAM2 scores for RP1-4 aligned with the SMCfs peptide and scores from the peptide microarray. The GLAM2-assigned similarity scores of the mimotopes to SMCfs led to a ranking of the top 4 peptides in the same order that they were ranked by the array absorption scoring described in section 3.2. For example, the RP1 peptide holds two motifs in common with the original antigen, exhibits the highest intensity on the peptide microarray, and scored the highest by the GLAM2 software. By contrast, the absorbance intensities obtained in the ELISA experiment did not yield rankings identical with the other readouts. This suggests that the array format may enable higher resolution detection of antibody-peptide interactions. Taking together, the sequence analysis of the specific array peptides showed the advantage of our high density random peptide array for identifying the epitopes of unknown antigen.

## Conclusion

The techniques demonstrated here could be inexpensively and generally applied for both mimotope and true-epitope discovery. We have demonstrated that non-natural sequence peptide arrays can be used to identify mimotopes of specific antibodies without knowing the sequence information of the primary epitope. This high density pepetide array platform is sensitive enough to identify at least two different specific antibodies to a simple peptide from the highly reactive antibodies which recognize KLH, a highly immunogenic antigen. The sequence analysis of the identified mimotopes can help identify the true B cell epitopes of an immunogen. Furthermore, the selected mimotopes can be used to purify this specific polyclonal antibody from the complex milieu of a serum sample for further analysis. In addition to be being a universal platform for the epitope exploration of any antibody, the non-natural sequence peptide array requires no assumptions or information relative to the true epitope of an immunogen, and therefore even mimotopes of newly mutated antigens can be discovered. As the SMCfs peptide was not an annotated, but frame-shift antigen, this method may allow discovery of the immunogenic epitopes of non-annotated antigens, such as novel mutations in tumor cells.

We suggest that mimotopes can be used to identify medically important antibodies and their natural targets. Additionally, mimotopes can be used as alternatives to a true epitope if they are found to carry advantageous properties. These may include greater stability, simpler production, or differential affinity relative to an autoantigen. These are all important attributes for successful drug or therapeutic development.

## Supporting Information

S1 Table Selective binding of anti-SMCfs serum to a set of 108 random sequence peptides displayed on a microarray. The intensities of all 10,000 peptides are available upon request.
(DOCX)

S2 Table GLAM4 analysis of the sequence similarity between the 108 selected peptides and KLH protein sequence. (DOCX)

S3 Table Changes in 108 random peptide binding intensities following anti-SMCfs antibody depletion of serum. The intensity of all 10,000 peptides are available upon request.
(DOCX)

S4 Table GLAM2 analysis of the sequence similarity between 108 selected array peptides and the SMCfs peptide. (DOCX)

## Abbreviations

BSA: Bovine serum albumin
HRP: Horse radish peroxidase
KLH: Keyhole limpet hemocyanin
PBS: Phosphate buffered saline
PBST: Phosphate buffered saline Tween-20
SMC1A: Structural maintenance chromosome 1A
SMC1Afs: Structural maintenance chromosome 1A frame-shift
SMCfs: A 17 amino acid SMC1A frameshift mutant
TBST: Tris Buffered Saline Tween-20
TMB: 3,3’,5,5’-Tetramethylbenzidine
TRIS: tris (hydroxymethyl) aminomethane
