# Supplementary material for: A General Method to Discover Epitopes from Sera
Source: PLoS One. 2016 Jun 14;11(6):e0157462. doi: 10.1371/journal.pone.0157462 (PMC4907474; doi:10.1371/journal.pone.0157462)
Supplement: S1 Table — The intensities of all 10,000 peptides are available upon request. (DOCX) [file pone.0157462.s001.docx]

| **Supplementary Table 1** | | | |  | | |  | | |  |  |
| --- | --- | --- | --- | --- | --- | --- | --- | --- | --- | --- | --- |
| Selective binding of anti-SMCfs serum to a set of 108 random sequence peptides displayed on a microarray. The intensities of all 10,000 peptides are available upon request. | | | | | | | | |  |  |  |
| Peptide | SMC1fs 1 | SMC1fs 2 | Naïve 1 | | Naïve 2 | Naïve 3 | |  |  |  |  |
| FPRKRNWWNTGPMREMNGSC | 65535 | 65535 | 618.5 | | 612 | 471 | |  |  |  |  |
| TISKYVMVEPMRQHEEWGSC | 65535 | 65535 | 341 | | 396 | 368 | |  |  |  |  |
| AVSHQEMNEGEQGPMREGSC | 65535 | 65535 | 654 | | 667.5 | 875 | |  |  |  |  |
| RVGEMPMREYDISGGSGGSC | 65535 | 65535 | 309.5 | | 377.5 | 292 | |  |  |  |  |
| TAFYRTLTKHEVDPGIAGSC | 65535 | 65535 | 412 | | 411 | 339.5 | |  |  |  |  |
| LGGLSPMRETVVWWHWHGSC | 65535 | 65535 | 350 | | 425.5 | 334 | |  |  |  |  |
| NDAGTIVIGHNQYLNGMGSC | 65535 | 65535 | 452.5 | | 429.5 | 385 | |  |  |  |  |
| RWRIIHGEWMLLKKWGHGSC | 65535 | 65535 | 6546.5 | | 5829 | 6312.5 | |  |  |  |  |
| HYNRYMVIIGNWGKQPIGSC | 65535 | 65535 | 2955 | | 2774 | 2039.5 | |  |  |  |  |
| KARWNGRNMTAPVYWRNGSC | 16752.5 | 17037.5 | 2813 | | 2451.5 | 2399 | |  |  |  |  |
| QRSWFSGKEPKFQRIWKGSC | 10009.5 | 10110.5 | 1800.5 | | 1742 | 1367.5 | |  |  |  |  |
| GYREILLLHHAQSRKVQGSC | 6352.5 | 6329 | 536.5 | | 385 | 440.5 | |  |  |  |  |
| DQMLMMQQQNTRPPRVFGSC | 4249 | 4314.5 | 633 | | 626 | 491.5 | |  |  |  |  |
| NPAWQAMTDILIGYNRPGSC | 7876 | 8151 | 1049.5 | | 1241.5 | 656.5 | |  |  |  |  |
| RWIYTHHLADRVRRKGPGSC | 10864 | 10555 | 1315.5 | | 1630.5 | 1139 | |  |  |  |  |
| VGLPAIGNRRRKFKRIIGSC | 9309 | 9096.5 | 1872.5 | | 1408.5 | 1648 | |  |  |  |  |
| EPKLWFKPRRGGYRHRHGSC | 13693.5 | 13602 | 2312.5 | | 2134.5 | 2601.5 | |  |  |  |  |
| WRWWFKRWRFRRKWHWFGSC | 12209 | 12402 | 794.5 | | 844.5 | 554 | |  |  |  |  |
| PMWLKTYHSSWYNSSHKGSC | 6110 | 6092.5 | 477 | | 501 | 404.5 | |  |  |  |  |
| HRFRFWKRWRKRRWFHKGSC | 7054 | 6900.5 | 2417 | | 2387 | 2150 | |  |  |  |  |
| MQMPSFYRGSLPDKHSTGSC | 2571 | 2647.5 | 545.5 | | 616.5 | 453.5 | |  |  |  |  |
| RHWRPKFRKFRWWRWHHGSC | 8279.5 | 8124 | 2184 | | 2380 | 2150 | |  |  |  |  |
| WWFKKWFKKFRHFPWHKGSC | 9841.5 | 9699 | 3801.5 | | 3943 | 4074 | |  |  |  |  |
| FVYRRGIVPTVGKVKRQGSC | 11002.5 | 11103 | 1889 | | 1709.5 | 1501 | |  |  |  |  |
| GGEKRRKNATKHEQWILGSC | 4840 | 4985 | 1431 | | 1148.5 | 1426.5 | |  |  |  |  |
| EYSMRFKWKWMKPGSFRGSC | 12745.5 | 12709.5 | 407 | | 541 | 437 | |  |  |  |  |
| GKIRFMSFMKGWNIHNIGSC | 17513.5 | 17660 | 1309 | | 1357 | 994.5 | |  |  |  |  |
| INVAGRRKYSIFSKERKGSC | 5880.5 | 5985 | 1528.5 | | 1254.5 | 1406.5 | |  |  |  |  |
| ERWDESQGMWWQVEPQWGSC | 3888.5 | 3853 | 1042.5 | | 994 | 1107.5 | |  |  |  |  |
| KQHKRDYDDSTENHSHTGSC | 8100 | 7881.5 | 873.5 | | 1133 | 731 | |  |  |  |  |
| MIGMTRHHGIVMPFGSHGSC | 11879.5 | 12205.5 | 1011.5 | | 1167 | 665.5 | |  |  |  |  |
| KNRWPAATRYHATIKQWGSC | 9302.5 | 9208 | 1310 | | 1454 | 1021.5 | |  |  |  |  |
| ETDSQQNYKYNKRDKRTGSC | 8359 | 8419 | 740.5 | | 857.5 | 603.5 | |  |  |  |  |
| EKSNDQHDNNQTDSRSEGSC | 6645.5 | 6727.5 | 1166 | | 1374 | 1037 | |  |  |  |  |
| MAPLAKILRERYVAKTPGSC | 5387.5 | 5438 | 1284.5 | | 1158 | 1098 | |  |  |  |  |
| WIIKHKDVAKKGTFAGKGSC | 65266 | 64956 | 1342 | | 1400.5 | 1834 | |  |  |  |  |
| PGKDRADWKHYGNYYPTGSC | 5271.5 | 5248 | 1810 | | 1823.5 | 1959.5 | |  |  |  |  |
| EDRFFMNDIKDRSMRFTGSC | 8499.5 | 8538.5 | 688 | | 493 | 467 | |  |  |  |  |
| ESHDQRTVQLKRQPIHWGSC | 4891.5 | 5004.5 | 920.5 | | 1073 | 725.5 | |  |  |  |  |
| KSHDTNEESSNRQDSNKGSC | 3984.5 | 4032 | 1188 | | 1004 | 1003.5 | |  |  |  |  |
| WKKLYDKFQQRLTHMADGSC | 2833 | 2896.5 | 683.5 | | 676 | 590 | |  |  |  |  |
| FIQTGNRRRVFQWGTNGGSC | 8221.5 | 7974.5 | 1068.5 | | 1307 | 798.5 | |  |  |  |  |
| KDKGVSPGHFHKMTWKFGSC | 8289.5 | 8218.5 | 1487.5 | | 1319.5 | 1219.5 | |  |  |  |  |
| IMLHPPWMLIQHTMWNQGSC | 5892 | 6046 | 437 | | 585 | 358 | |  |  |  |  |
| QIGSYNWLVHAPFAKLMGSC | 2669 | 2683.5 | 421.5 | | 445 | 358.5 | |  |  |  |  |
| GMTKHYYQYPDSKKTLKGSC | 5466 | 5472.5 | 1027 | | 1114.5 | 1065 | |  |  |  |  |
| VWGKGGMYEAHYRRNGEGSC | 2731.5 | 2662.5 | 464 | | 443.5 | 355 | |  |  |  |  |
| LRKISRGIWGMREAGEFGSC | 4618 | 4487 | 1009.5 | | 798.5 | 786 | |  |  |  |  |
| WNHMDVDNFHYVETYRYGSC | 4552 | 4449.5 | 738.5 | | 870.5 | 668.5 | |  |  |  |  |
| VLAIILIIVLIAIVLIIGSC | 6140.5 | 6252 | 506 | | 586.5 | 422 | |  |  |  |  |
| YFIEVRWSTVSITIHHKGSC | 4320.5 | 4364.5 | 397.5 | | 464 | 316.5 | |  |  |  |  |
| MNSGVRWLHSYYKESHMGSC | 2117.5 | 2120.5 | 545.5 | | 534 | 504.5 | |  |  |  |  |
| IFRYVKDFAKADTHKWMGSC | 5985 | 5816 | 845.5 | | 939 | 596 | |  |  |  |  |
| YWVDSWPHFADNLTTRLGSC | 2297 | 2327.5 | 656 | | 657.5 | 581.5 | |  |  |  |  |
| RNHDESSRNKNHYKNDYGSC | 2211 | 2149 | 545 | | 538.5 | 402 | |  |  |  |  |
| SGMHIVLRNGKMFEYSMGSC | 2026.5 | 1970 | 560.5 | | 620 | 495 | |  |  |  |  |
| LVWLMSTMHGGDNQIHDGSC | 1984 | 2020 | 400.5 | | 336.5 | 292 | |  |  |  |  |
| HHMFMMEWMWSALHPGHGSC | 1353.5 | 1345 | 292 | | 333.5 | 294.5 | |  |  |  |  |
| TGILKPKDDPMLWSWVMGSC | 1010.5 | 1026 | 540 | | 543.5 | 502.5 | |  |  |  |  |
| SKPKRVMRNWNSQSWDPGSC | 2149 | 2177 | 371.5 | | 417 | 322 | |  |  |  |  |
| MHSDVNSIRQRLYKNKMGSC | 3334 | 3267.5 | 778.5 | | 784 | 618.5 | |  |  |  |  |
| NGYRINDHTPNQKPYSYGSC | 1699.5 | 1737 | 568.5 | | 642 | 540.5 | |  |  |  |  |
| VFQTYHWVNSNALLYNPGSC | 1743.5 | 1769 | 381 | | 370.5 | 332.5 | |  |  |  |  |
| LPHYPYQFMPWFSGWYWGSC | 1355.5 | 1380 | 551.5 | | 517 | 497.5 | |  |  |  |  |
| KSHDLGNDRSMKFRNRGGSC | 4237 | 4164 | 906.5 | | 841 | 743 | |  |  |  |  |
| TLNKRRSWRDGFTADEYGSC | 1299 | 1309 | 411.5 | | 383 | 369 | |  |  |  |  |
| DMTRVESQQTHTPVQIAGSC | 4086 | 4041 | 579 | | 729.5 | 562 | |  |  |  |  |
| DTGDMNPGYNHIWRTRNGSC | 844 | 834 | 346 | | 306.5 | 319 | |  |  |  |  |
| HPTKMHQPHHLYWSLVQGSC | 3697 | 3627 | 497 | | 532.5 | 417 | |  |  |  |  |
| PMHEVIQWYTQADMHADGSC | 552.5 | 554.5 | 335.5 | | 345 | 338 | |  |  |  |  |
| KMNGQGMKYWHWSRAQYGSC | 4942.5 | 4966.5 | 506 | | 445.5 | 378.5 | |  |  |  |  |
| VGPYDNQNYTIWRYTHFGSC | 893.5 | 890 | 355.5 | | 365.5 | 346.5 | |  |  |  |  |
| WDYADINRYTAQEHTHTGSC | 652.5 | 658 | 301 | | 301 | 286.5 | |  |  |  |  |
| HTDFTVYMSFDHPGKGQGSC | 2011 | 2073 | 412.5 | | 460 | 349 | |  |  |  |  |
| TNWMKHIIPNVFAFVNNGSC | 1368.5 | 1352 | 369 | | 395.5 | 324.5 | |  |  |  |  |
| WGIYASWKHDNPGSMMYGSC | 1440.5 | 1471.5 | 462 | | 402.5 | 446 | |  |  |  |  |
| HNVIEVERKGQKMQGQFGSC | 509 | 502 | 289 | | 277 | 277 | |  |  |  |  |
| QTERTESWHGEVPIIDLGSC | 516.5 | 512.5 | 324 | | 309 | 315 | |  |  |  |  |
| RMHPRLSAFQWNNDNSIGSC | 1661.5 | 1613.5 | 407.5 | | 422 | 341 | |  |  |  |  |
| DGDTVWRLPKSRFVGVIGSC | 1051 | 1073 | 396.5 | | 382 | 356 | |  |  |  |  |
| QSQYDQSNESESNSYTDGSC | 2233.5 | 2274.5 | 492 | | 496.5 | 372.5 | |  |  |  |  |
| AEQNIQSSGMHAMRDRDGSC | 486.5 | 480 | 270.5 | | 255 | 260 | |  |  |  |  |
| ALGLMLALYSHGGKWPDGSC | 1605.5 | 1596 | 461 | | 490.5 | 439 | |  |  |  |  |
| KHEMWNWVFLTVNKERVGSC | 2602 | 2585.5 | 483.5 | | 420 | 452.5 | |  |  |  |  |
| TYKMVRVGHFYSYVAFRGSC | 4358 | 4283 | 570 | | 531 | 457 | |  |  |  |  |
| EREIRPNQVWMENIWFMGSC | 796 | 809 | 414.5 | | 446 | 431 | |  |  |  |  |
| PTYHIALIDELGAQYSHGSC | 993.5 | 1019.5 | 360.5 | | 413 | 387.5 | |  |  |  |  |
| KLNGWTIPAHIEMHFHVGSC | 7129 | 7100 | 489.5 | | 462 | 442.5 | |  |  |  |  |
| RFTWFGMWAAMFKPRPQGSC | 2713.5 | 2637.5 | 676 | | 697 | 513 | |  |  |  |  |
| AWNGQTIEREHMLGWPVGSC | 1468.5 | 1436 | 551.5 | | 514 | 458 | |  |  |  |  |
| KHTAFHNHETVRVHSWFGSC | 2664.5 | 2633 | 478 | | 511 | 431 | |  |  |  |  |
| NHKAVSNHHAYGDYFWSGSC | 1908 | 1870 | 773 | | 785.5 | 871 | |  |  |  |  |
| AFLWMTNISPTIFYSARGSC | 3929 | 3982 | 487 | | 515.5 | 405.5 | |  |  |  |  |
| IVKYWSFNQFRIHRQWSGSC | 2227.5 | 2169.5 | 649.5 | | 615 | 515.5 | |  |  |  |  |
| HFSKESWKERLVSTAVGGSC | 1706.5 | 1662.5 | 378.5 | | 430.5 | 331 | |  |  |  |  |
| QLHHWMSSDWAGPFQHVGSC | 2164 | 2157.5 | 377 | | 417 | 384 | |  |  |  |  |
| RSALTGKGRLAEKTEKAGSC | 3084.5 | 3132.5 | 966.5 | | 880.5 | 807 | |  |  |  |  |
| WTGLSEGKERGRGRLWLGSC | 7400 | 7363 | 757.5 | | 942 | 645 | |  |  |  |  |
| FWPNNMEWIILHGFIWLGSC | 2901.5 | 2948.5 | 383.5 | | 445 | 328.5 | |  |  |  |  |
| HKVRSMAYHLVFFEEDEGSC | 1608 | 1620 | 494 | | 460.5 | 442.5 | |  |  |  |  |
| MHAHNPLYIHLNYLDHPGSC | 3218.5 | 3232 | 475.5 | | 564.5 | 551.5 | |  |  |  |  |
| WWGREGWEREKRTTWLKGSC | 6543 | 6367 | 1027 | | 1239 | 866 | |  |  |  |  |
| EHGQPQPSHDWYGVFRYGSC | 65535 | 65535 | 524 | | 617.5 | 459.5 | |  |  |  |  |
| AMYKYHRPIATRMLPLFGSC | 65535 | 65535 | 675.5 | | 911.5 | 704.5 | |  |  |  |  |
| EGNGWSGVNGNLFPRQGGSC | 65535 | 65535 | 586 | | 550 | 500.5 | |  |  |  |  |
| ESAHSLWFGWRSVRHFDGSC | 65535 | 65535 | 1134 | | 1254.5 | 1024 | |  |  |  |  |
| QFSKGQTIIFVPQKFKEGSC | 54790.5 | 54853 | 739 | | 811.5 | 821.5 | |  |  |  |  |
| EGWHALLQFARDNWKPWGSC | 65535 | 65535 | 583.5 | | 393.5 | 472 | |  |  |  |  |
