# Supplementary material for: A General Method to Discover Epitopes from Sera
Source: PLoS One. 2016 Jun 14;11(6):e0157462. doi: 10.1371/journal.pone.0157462 (PMC4907474; doi:10.1371/journal.pone.0157462)
Supplement: S2 Table — (DOCX) [file pone.0157462.s002.docx]

**Supplementary Table 2.** GLAM2 analysis of the sequence similarity between the 108 selected peptides and KLH protein sequence.

| Peptide | Glam2Score | Matching KLH Sequence | Matching Peptide Sequence |
| --- | --- | --- | --- |
| KMNGQGMKYWHWSRAQY | 17.7875 | GLPYWDWTR | GMKYWHWSR |
| EHGQPQPSHDWYGVFRY | 17.6324 | KHNLPQDSFDYQNRFRY | EHGQPQPSHDWYGVFRY |
| EYSMRFKWKWMKPGSFR | 15.2567 | WDWLKPQS | WKWMKPGS |
| GYREILLLHHAQSRKVQ | 15.026 | YDPLFWLHHANTDRI | YREILLLHHAQSRKV |
| LVWLMSTMHGGDNQIHD | 14.604 | HGGDTSGHD | HGGDNQIHD |
| PTYHIALIDELGAQYSH | 14.5332 | TFHMRIIDTSGKQ | TYHIALIDELGAQ |
| NGYRINDHTPNQKPYSY | 14.3939 | YNLNDHT | YRINDHT |
| HPTKMHQPHHLYWSLVQ | 14.2909 | HHEKHHEDHH | HPTKMHQPHH |
| WRWWFKRWRFRRKWHWF | 14.2808 | KWGFYRAYHF | RWRFRRKWHW |
| NHKAVSNHHAYGDYFWS | 14.1822 | HHANTDRIW | HHAYGDYFW |
| TNWMKHIIPNVFAFVNN | 14.0856 | WRNKVMPNPFA | WMKHIIPNVFA |
| AFLWMTNISPTIFYSAR | 13.9892 | PTIIYSA | PTIFYSA |
| LGGLSPMRETVVWWHWH | 13.9693 | VFPHWH | VWWHWH |
| MHAHNPLYIHLNYLDHP | 13.7141 | LHAEDHFYIDYEVFD | MHAHNPLYIHLNYLD |
| QRSWFSGKEPKFQRIWK | 13.6171 | TWVGGKEP | SWFSGKEP |
| KDKGVSPGHFHKMTWKF | 13.5889 | GGEHEMPWSF | GHFHKMTWKF |
| WNHMDVDNFHYVETYRY | 13.5656 | DSFDYQNRFRY | DNFHYVETYRY |
| WGIYASWKHDNPGSMMY | 13.548 | WGFYRAYHFD | WGIYASWKHD |
| QLHHWMSSDWAGPFQHV | 13.3689 | DWTEPMTHI | DWAGPFQHV |
| WWFKKWFKKFRHFPWHK | 13.3602 | WTKRIEHLP | WFKKFRHFP |
| VLAIILIIVLIAIVLII | 13.2756 | MLSVRLLIVVLAL | VLAIILIIVLIAI |
| WDYADINRYTAQEHTHT | 13.2214 | WAYERLYRFDITETVH | WDYADINRYTAQEHTH |
| HRFRFWKRWRKRRWFHK | 13.1839 | RIWAIWQDLQRFRK | RFWKRWRKRRWFHK |
| VGPYDNQNYTIWRYTHF | 13.0463 | DKEYYDVWR | DNQNYTIWR |
| HHMFMMEWMWSALHPGH | 13.0225 | HHTRLMEGILDAL | HHMFMMEWMWSAL |
| LPHYPYQFMPWFSGWYW | 12.8936 | PWFDG | PWFSG |
| DTGDMNPGYNHIWRTRN | 12.6534 | GIDHIVRNR | GYNHIWRTR |
| RWIYTHHLADRVRRKGP | 12.603 | LYTVQFEDSLKRHG | IYTHHLADRVRRKG |
| AMYKYHRPIATRMLPLF | 12.5919 | FYKYH | MYKYH |
| RMHPRLSAFQWNNDNSI | 12.5036 | LHQKMEPFSWE.DNDI | MHPRLSAFQWNNDNSI |
| PGKDRADWKHYGNYYPT | 12.4434 | EDTFDYQKFGYIYDT | KDRADWKHYGNYYPT |
| RFTWFGMWAAMFKPRPQ | 12.3734 | LGSYTAMFK | FGMWAAMFK |
| IMLHPPWMLIQHTMWNQ | 12.3401 | IMFHP | IMLHP |
| MHSDVNSIRQRLYKNKM | 12.3108 | HHEAEELRDALYK | HSDVNSIRQRLYK |
| ESAHSLWFGWRSVRHFD | 12.3098 | IWAIWQSLQRF | LWFGWRSVRHF |
| FWPNNMEWIILHGFIWL | 12.309 | FLLHGF | IILHGF |
| EGWHALLQFARDNWKPW | 12.2971 | WHRLLTVQSEN | WHALLQFARDN |
| WIIKHKDVAKKGTFAGK | 12.2596 | HKQTQHDRTFAG | HKDVAKKGTFAG |
| RHWRPKFRKFRWWRWHH | 12.2473 | WHH | WHH |
| GGEKRRKNATKHEQWIL | 12.2135 | GGKHHEKHHEDHHEDIL | GGEKRRKNATKHEQWIL |
| TAFYRTLTKHEVDPGIA | 12.1953 | FERALKKHGSHLGI | FYRTLTKHEVDPGI |
| KLNGWTIPAHIEMHFHV | 12.1616 | HAEDHFYI | HIEMHFHV |
| ETDSQQNYKYNKRDKRT | 12.1167 | NYHYDNPDVR | NYKYNKRDKR |
| PMWLKTYHSSWYNSSHK | 12.1063 | KTYHNS | KTYHSS |
| EPKLWFKPRRGGYRHRH | 12.0974 | GGYEH | GGYRH |
| TGILKPKDDPMLWSWVM | 12.0854 | WDWTM | WSWVM |
| GKIRFMSFMKGWNIHNI | 12.0499 | GWSIRGI | GWNIHNI |
| EDRFFMNDIKDRSMRFT | 12.0367 | DRVFKYDITEK | DRFFMNDIKDR |
| TYKMVRVGHFYSYVAFR | 12.0317 | LRDGHYY | VRVGHFY |
| WKKLYDKFQQRLTHMAD | 11.9823 | WHRLYTK | WKKLYDK |
| HYNRYMVIIGNWGKQPI | 11.9524 | HWHRLYVVVVEN | HYNR.YMVIIGN |
| FIQTGNRRRVFQWGTNG | 11.9107 | NRDRLFQ | NRRRVFQ |
| INVAGRRKYSIFSKERK | 11.8747 | LGGRAKYSL | VAGRRKYSI |
| EKSNDQHDNNQTDSRSE | 11.7467 | KCNDSHNN | KSNDQHDN |
| ALGLMLALYSHGGKWPD | 11.6593 | MALLSHG | LALYSHG |
| NDAGTIVIGHNQYLNGM | 11.6506 | INHNQF | IGHNQY |
| TLNKRRSWRDGFTADEY | 11.638 | THNAIHSWTGGLT | TLNKRRSWRDGFT |
| HTDFTVYMSFDHPGKGQ | 11.6148 | SDFTFHMRIIDTSGK | TDFTVYMSF.DHPGK |
| DQMLMMQQQNTRPPRVF | 11.4566 | QLDHMLQERKRHDRVF | QMLMMQQQNTRPPRVF |
| RNHDESSRNKNHYKNDY | 11.4442 | DDNDRNDDH | DESSRNKNH |
| KQHKRDYDDSTENHSHT | 11.4397 | DDETREHS | DDSTENHS |
| YWVDSWPHFADNLTTRL | 11.4135 | WFDGHIDTVDKTTTR | WVDSWPHFADNLTTR |
| QIGSYNWLVHAPFAKLM | 11.36 | VVNNPFAK | LVHAPFAK |
| RVGEMPMREYDISGGSG | 11.3517 | GENALRKHGFTGG | GEMPMREYDISGG |
| KHTAFHNHETVRVHSWF | 11.3346 | HNWF | HSWF |
| KNRWPAATRYHATIKQW | 11.3166 | HNTIHAW | HATIKQW |
| IVKYWSFNQFRIHRQWS | 11.1577 | IPYWDWTQ | VKYWSFNQ |
| DGDTVWRLPKSRFVGVI | 11.1369 | WTLPRSEL | WRLPKSRF |
| LRKISRGIWGMREAGEF | 11.1097 | VRDVNEAIFQQTKFGEF | LRKISRGIWGMREAGEF |
| YFIEVRWSTVSITIHHK | 11.0951 | FEVQFEVVHNAIHY | IEVRWSTVSITIHH |
| VWGKGGMYEAHYRRNGE | 11.09 | WGFDRMYK | WGKGGMYE |
| TISKYVMVEPMRQHEEW | 11.0142 | MMTKPMR | VMVEPMR |
| WWGREGWEREKRTTWLK | 10.9539 | YWGLPYWD | WWGREGWE |
| WTGLSEGKERGRGRLWL | 10.8947 | GLDRLWI | GRGRLWL |
| AVSHQEMNEGEQGPMRE | 10.7913 | LSPEEMNE | VSHQEMNE |
| KHEMWNWVFLTVNKERV | 10.7617 | WDWL | WNWV |
| HKVRSMAYHLVFFEEDE | 10.7592 | AYDPIFF | AYHLVFF |
| MIGMTRHHGIVMPFGSH | 10.7525 | LVIPLGS | IVMPFGS |
| MNSGVRWLHSYYKESHM | 10.6971 | VSSLHNYIKQ | VRWLHSYYKE |
| DMTRVESQQTHTPVQIA | 10.6431 | HTPLQ | HTPVQ |
| GMTKHYYQYPDSKKTLK | 10.6165 | YQYDD | YQYPD |
| VFQTYHWVNSNALLYNP | 10.5617 | HILNEPSLLFVP | HWVNSNALLYNP |
| KSHDLGNDRSMKFRNRG | 10.5588 | KEMKWGFDRVFKF | KSHDLGNDRSMKF |
| ESHDQRTVQLKRQPIHW | 10.5226 | LKAQSIH | LKRQPIH |
| FVYRRGIVPTVGKVKRQ | 10.5177 | FTFHMRIIDTSGK | FVYRRGIVPTVGK |
| FPRKRNWWNTGPMREMN | 10.4946 | NWDWTMPM | NWWNTGPM |
| SKPKRVMRNWNSQSWDP | 10.4704 | NSRSQTFDP | NWNSQSWDP |
| HNVIEVERKGQKMQGQF | 10.466 | HNIEELE | HNVIEVE |
| EGNGWSGVNGNLFPRQG | 10.3156 | VNGTVLP | VNGNLFP |
| RWRIIHGEWMLLKKWGH | 10.307 | LKKYDH | LKKWGH |
| MQMPSFYRGSLPDKHST | 10.1416 | MPSF | MPSF |
| SGMHIVLRNGKMFEYSM | 10.0958 | VFEYS | MFEYS |
| KSHDTNEESSNRQDSNK | 9.95936 | HPFNYESVNNDD | HDTNEESSNRQD |
| IFRYVKDFAKADTHKWM | 9.93029 | HIKDIAGAE | YVKDFAKAD |
| QFSKGQTIIFVPQKFKE | 9.86272 | RGTRIMFHP | KGQTIIFVP |
| ERWDESQGMWWQVEPQW | 9.75748 | EPKW | EPQW |
| QTERTESWHGEVPIIDL | 9.72981 | PVVDL | PIIDL |
| PMHEVIQWYTQADMHAD | 9.71627 | MHNTIHY | MHEVIQW |
| AWNGQTIEREHMLGWPV | 9.64449 | ISLEHM | IEREHM |
| AEQNIQSSGMHAMRDRD | 9.61643 | MHWAYDRN | MHAMRDRD |
| MAPLAKILRERYVAKTP | 9.61584 | LIPHASVIRE | MAPLAKILRE |
| KARWNGRNMTAPVYWRN | 9.58519 | WRN | WRN |
| EREIRPNQVWMENIWFM | 9.37278 | LDKIWII | MENIWFM |
| VGLPAIGNRRRKFKRII | 9.36366 | VGLP | VGLP |
| NPAWQAMTDILIGYNRP | 9.24986 | LVGGNEP | LIGYNRP |
| RSALTGKGRLAEKTEKA | 9.20124 | SLLGGKGK | SALTGKGR |
| HFSKESWKERLVSTAVG | 8.94194 | HVDDEMW | HFSKESW |
| QSQYDQSNESESNSYTD | 7.92837 | QYD | QYD |
