# Supplementary material for: A General Method to Discover Epitopes from Sera
Source: PLoS One. 2016 Jun 14;11(6):e0157462. doi: 10.1371/journal.pone.0157462 (PMC4907474; doi:10.1371/journal.pone.0157462)
Supplement: S3 Table — The intensity of all 10,000 peptides are available upon request. (DOCX) [file pone.0157462.s003.docx]

| **Supplementary Table 3** |  |  |  |  |  |  |  |
| --- | --- | --- | --- | --- | --- | --- | --- |
| Changes in 108 random peptide binding intensities following anti-SMCfs antibody depletion of serum. The intensity of all 10,000 peptides are available upon request. | | | | | | | |
| Peptide | SMCfs | 11x SMCfs absorbed | 11x SMCfs absorbed 2 | 20x SMCfs absorbed | 20x SMCfs absorbed 2 | 20x CP1 absorbed | 20x CP1 absorbed 2 |
| FPRKRNWWNTGPMREMNGSC | 65535 | 790 | 30259 | 972.5 | 94 | 65535 | 63883.5 |
| TISKYVMVEPMRQHEEWGSC | 65535 | 932 | 16644 | 347.5 | 84 | 65535 | 20810.5 |
| AVSHQEMNEGEQGPMREGSC | 65535 | 836.5 | 10167.5 | 282 | 87.5 | 65535 | 13924.5 |
| RVGEMPMREYDISGGSGGSC | 65535 | 542.5 | 8433 | 343 | 85.5 | 65535 | 12844.5 |
| TAFYRTLTKHEVDPGIAGSC | 65535 | 706 | 37020.5 | 296 | 80 | 65535 | 10942.5 |
| LGGLSPMRETVVWWHWHGSC | 47777 | 547 | 1096 | 433.5 | 88 | 53293 | 4685 |
| NDAGTIVIGHNQYLNGMGSC | 65535 | 549.5 | 4241 | 410.5 | 93 | 55892.5 | 4122.5 |
| RWRIIHGEWMLLKKWGHGSC | 65535 | 65301.5 | 61543.5 | 41600.5 | 968 | 46867 | 20137 |
| HYNRYMVIIGNWGKQPIGSC | 65535 | 65535 | 65535 | 60306 | 727.5 | 52994 | 25698 |
| KARWNGRNMTAPVYWRNGSC | 12664.5 | 6562.5 | 5418.5 | 14689 | 239 | 6466 | 10583.5 |
| QRSWFSGKEPKFQRIWKGSC | 8960.5 | 2958 | 3210.5 | 3466.5 | 142.5 | 2442 | 4326.5 |
| GYREILLLHHAQSRKVQGSC | 10628.5 | 4656 | 3817 | 4327.5 | 343 | 5697 | 4326.5 |
| DQMLMMQQQNTRPPRVFGSC | 1470 | 1449 | 857 | 633 | 82.5 | 3878 | 2369 |
| NPAWQAMTDILIGYNRPGSC | 3026.5 | 1177.5 | 803 | 335.5 | 169.5 | 2004.5 | 2640.5 |
| RWIYTHHLADRVRRKGPGSC | 12066 | 3072.5 | 2850 | 295.5 | 166.5 | 2176 | 2300.5 |
| VGLPAIGNRRRKFKRIIGSC | 9877 | 5862 | 5427.5 | 4857 | 235.5 | 5281 | 3484 |
| EPKLWFKPRRGGYRHRHGSC | 15309 | 5673 | 5149.5 | 4515 | 227.5 | 3925.5 | 3381.5 |
| WRWWFKRWRFRRKWHWFGSC | 1878 | 869 | 607.5 | 5245 | 279 | 4793.5 | 3277 |
| PMWLKTYHSSWYNSSHKGSC | 2317.5 | 642 | 601.5 | 1625.5 | 92.5 | 1572.5 | 1911 |
| HRFRFWKRWRKRRWFHKGSC | 5365.5 | 3673.5 | 4060 | 2091.5 | 124 | 3459 | 1756 |
| MQMPSFYRGSLPDKHSTGSC | 1733.5 | 3027 | 1957 | 2019 | 138.5 | 2294.5 | 1958 |
| RHWRPKFRKFRWWRWHHGSC | 3318.5 | 2882.5 | 3164 | 2460 | 195.5 | 3036 | 2020 |
| WWFKKWFKKFRHFPWHKGSC | 3238 | 2077.5 | 2132 | 1226 | 137 | 3003 | 1349.5 |
| FVYRRGIVPTVGKVKRQGSC | 6173.5 | 2361 | 1957.5 | 3108 | 203.5 | 3045.5 | 2218 |
| GGEKRRKNATKHEQWILGSC | 3607 | 1234.5 | 1433 | 1463 | 108.5 | 2394 | 1444.5 |
| EYSMRFKWKWMKPGSFRGSC | 1812.5 | 729 | 828 | 3174.5 | 217.5 | 4142.5 | 1963 |
| GKIRFMSFMKGWNIHNIGSC | 14993 | 5334.5 | 5499.5 | 5902 | 187 | 5596.5 | 2749.5 |
| INVAGRRKYSIFSKERKGSC | 8087 | 2148.5 | 2037 | 2673 | 123.5 | 2788.5 | 1758 |
| ERWDESQGMWWQVEPQWGSC | 6289.5 | 4080.5 | 2186.5 | 954 | 181.5 | 3774.5 | 734.5 |
| KQHKRDYDDSTENHSHTGSC | 6913 | 4212.5 | 3123.5 | 2362 | 220 | 2438 | 1677 |
| MIGMTRHHGIVMPFGSHGSC | 9070.5 | 4203 | 2580 | 2748 | 164.5 | 4128 | 1353 |
| KNRWPAATRYHATIKQWGSC | 13476.5 | 4135 | 4006.5 | 2515 | 200.5 | 3147 | 1515 |
| ETDSQQNYKYNKRDKRTGSC | 9248 | 2445.5 | 2433.5 | 2092 | 149.5 | 2249.5 | 1431.5 |
| EKSNDQHDNNQTDSRSEGSC | 5450.5 | 3313.5 | 1854.5 | 2224 | 145.5 | 2107 | 1450 |
| MAPLAKILRERYVAKTPGSC | 4420.5 | 1720 | 1505.5 | 1247 | 97.5 | 2022 | 888.5 |
| WIIKHKDVAKKGTFAGKGSC | 54289.5 | 47743.5 | 16282 | 8782.5 | 1499 | 11211.5 | 4757.5 |
| PGKDRADWKHYGNYYPTGSC | 6572.5 | 4565 | 2378.5 | 3328.5 | 131 | 3572 | 1486 |
| EDRFFMNDIKDRSMRFTGSC | 7140 | 9935 | 3231.5 | 2951.5 | 123 | 5834 | 817.5 |
| ESHDQRTVQLKRQPIHWGSC | 5294 | 5085 | 1936 | 1325.5 | 103 | 1757 | 914 |
| KSHDTNEESSNRQDSNKGSC | 3343 | 1230.5 | 997 | 1347.5 | 111.5 | 1251 | 1046 |
| WKKLYDKFQQRLTHMADGSC | 3333.5 | 1112.5 | 729.5 | 555 | 94 | 1830 | 545.5 |
| FIQTGNRRRVFQWGTNGGSC | 4603.5 | 2233 | 1591 | 3980.5 | 132 | 3181 | 1794.5 |
| KDKGVSPGHFHKMTWKFGSC | 3854 | 2143.5 | 1027.5 | 2239 | 94.5 | 2633.5 | 1044.5 |
| IMLHPPWMLIQHTMWNQGSC | 983.5 | 613.5 | 520 | 856 | 85 | 1231 | 725 |
| QIGSYNWLVHAPFAKLMGSC | 1855 | 535.5 | 549 | 550 | 136.5 | 847.5 | 765 |
| GMTKHYYQYPDSKKTLKGSC | 2075.5 | 713 | 744.5 | 814 | 111.5 | 695.5 | 843 |
| VWGKGGMYEAHYRRNGEGSC | 1359 | 784 | 609.5 | 1367 | 88.5 | 2627.5 | 568 |
| LRKISRGIWGMREAGEFGSC | 5801 | 1540.5 | 2712.5 | 1156 | 95.5 | 2564 | 480 |
| WNHMDVDNFHYVETYRYGSC | 7320 | 3737.5 | 2744.5 | 288.5 | 97 | 1538 | 329 |
| VLAIILIIVLIAIVLIIGSC | 7022 | 2960 | 2316.5 | 1691.5 | 132 | 1452.5 | 980 |
| YFIEVRWSTVSITIHHKGSC | 3106.5 | 1561.5 | 1187 | 842 | 110.5 | 1105.5 | 619.5 |
| MNSGVRWLHSYYKESHMGSC | 2842 | 1226 | 744.5 | 647.5 | 100 | 1369.5 | 417.5 |
| IFRYVKDFAKADTHKWMGSC | 4694 | 1293 | 1519.5 | 2340.5 | 93 | 2720.5 | 831.5 |
| YWVDSWPHFADNLTTRLGSC | 4867.5 | 2796.5 | 1441 | 1230 | 100 | 1379.5 | 638 |
| RNHDESSRNKNHYKNDYGSC | 3765.5 | 693 | 668 | 500 | 86.5 | 1073 | 345.5 |
| SGMHIVLRNGKMFEYSMGSC | 2421.5 | 1103 | 808.5 | 648.5 | 84 | 1495.5 | 299.5 |
| LVWLMSTMHGGDNQIHDGSC | 2946 | 1438.5 | 835 | 477 | 95 | 1268 | 288.5 |
| HHMFMMEWMWSALHPGHGSC | 2602.5 | 1094 | 934.5 | 798 | 151 | 941.5 | 567 |
| TGILKPKDDPMLWSWVMGSC | 1172 | 767 | 535.5 | 333 | 83.5 | 758.5 | 255.5 |
| SKPKRVMRNWNSQSWDPGSC | 3814 | 796 | 1064 | 1338 | 108 | 1644 | 537.5 |
| MHSDVNSIRQRLYKNKMGSC | 3759 | 1232 | 781.5 | 628 | 88.5 | 1224 | 280 |
| NGYRINDHTPNQKPYSYGSC | 2524.5 | 1856.5 | 1164 | 506.5 | 98 | 1122.5 | 257 |
| VFQTYHWVNSNALLYNPGSC | 1090.5 | 643 | 529 | 500.5 | 114 | 815 | 320 |
| LPHYPYQFMPWFSGWYWGSC | 1347.5 | 944 | 671 | 540 | 90.5 | 1010 | 246.5 |
| KSHDLGNDRSMKFRNRGGSC | 3259 | 1034 | 790.5 | 737.5 | 90.5 | 1304 | 260 |
| TLNKRRSWRDGFTADEYGSC | 1014.5 | 748 | 670.5 | 553 | 84 | 1299 | 162.5 |
| DMTRVESQQTHTPVQIAGSC | 1192.5 | 666.5 | 562 | 511.5 | 172 | 824 | 408.5 |
| DTGDMNPGYNHIWRTRNGSC | 993 | 690.5 | 515.5 | 410 | 89.5 | 711.5 | 231 |
| HPTKMHQPHHLYWSLVQGSC | 2395.5 | 945 | 779 | 724.5 | 87 | 1103 | 259 |
| PMHEVIQWYTQADMHADGSC | 716 | 558.5 | 493 | 295.5 | 82 | 768.5 | 130.5 |
| KMNGQGMKYWHWSRAQYGSC | 3454 | 686 | 817.5 | 2167 | 101.5 | 2072.5 | 686.5 |
| VGPYDNQNYTIWRYTHFGSC | 1103 | 636.5 | 521.5 | 415.5 | 89 | 947 | 153 |
| WDYADINRYTAQEHTHTGSC | 1461.5 | 703.5 | 619 | 415.5 | 82.5 | 921.5 | 146 |
| HTDFTVYMSFDHPGKGQGSC | 16612 | 8919 | 6250.5 | 468 | 82.5 | 932.5 | 165.5 |
| TNWMKHIIPNVFAFVNNGSC | 2147 | 685.5 | 571.5 | 431.5 | 98.5 | 716 | 223.5 |
| WGIYASWKHDNPGSMMYGSC | 2302 | 1348.5 | 1102 | 585 | 90 | 1143 | 180.5 |
| HNVIEVERKGQKMQGQFGSC | 789.5 | 407 | 380 | 267 | 77 | 658.5 | 120.5 |
| QTERTESWHGEVPIIDLGSC | 678.5 | 548.5 | 509.5 | 302.5 | 85.5 | 744 | 123 |
| RMHPRLSAFQWNNDNSIGSC | 1304 | 586 | 584.5 | 436.5 | 85 | 867.5 | 148.5 |
| DGDTVWRLPKSRFVGVIGSC | 1297.5 | 739.5 | 1134 | 386.5 | 88.5 | 759 | 156 |
| QSQYDQSNESESNSYTDGSC | 3689.5 | 1058 | 946.5 | 762 | 95 | 934.5 | 290 |
| AEQNIQSSGMHAMRDRDGSC | 853.5 | 564 | 469 | 298 | 88.5 | 664 | 134.5 |
| ALGLMLALYSHGGKWPDGSC | 2323.5 | 1586 | 1017.5 | 535.5 | 96.5 | 846.5 | 188.5 |
| KHEMWNWVFLTVNKERVGSC | 1864.5 | 1463.5 | 729.5 | 477 | 89 | 893.5 | 136 |
| TYKMVRVGHFYSYVAFRGSC | 6776.5 | 1257.5 | 1032.5 | 882.5 | 175.5 | 1103.5 | 426 |
| EREIRPNQVWMENIWFMGSC | 1444 | 893.5 | 581.5 | 426.5 | 92 | 621.5 | 176 |
| PTYHIALIDELGAQYSHGSC | 1277 | 562.5 | 512 | 375.5 | 86.5 | 605.5 | 140.5 |
| KLNGWTIPAHIEMHFHVGSC | 3677.5 | 936.5 | 917.5 | 1505 | 85.5 | 2023 | 314.5 |
| RFTWFGMWAAMFKPRPQGSC | 2646.5 | 910.5 | 857.5 | 603.5 | 87.5 | 846 | 177 |
| AWNGQTIEREHMLGWPVGSC | 1919.5 | 1593.5 | 979.5 | 951 | 87.5 | 1305.5 | 203 |
| KHTAFHNHETVRVHSWFGSC | 1483 | 535 | 482.5 | 463.5 | 92 | 673.5 | 134.5 |
| NHKAVSNHHAYGDYFWSGSC | 2241.5 | 1170 | 883 | 805.5 | 95 | 1142.5 | 176.5 |
| AFLWMTNISPTIFYSARGSC | 4374 | 2553 | 1431.5 | 1124.5 | 89 | 1391 | 234 |
| IVKYWSFNQFRIHRQWSGSC | 3362 | 759 | 904.5 | 746.5 | 91 | 909.5 | 174.5 |
| HFSKESWKERLVSTAVGGSC | 1115 | 471 | 419 | 572 | 94.5 | 767 | 136 |
| QLHHWMSSDWAGPFQHVGSC | 1521 | 619 | 736 | 769 | 90.5 | 999 | 151 |
| RSALTGKGRLAEKTEKAGSC | 4162 | 2214 | 1240 | 1039.5 | 289.5 | 1006 | 605 |
| WTGLSEGKERGRGRLWLGSC | 4702 | 985.5 | 1214.5 | 3871.5 | 165 | 3264 | 1063.5 |
| FWPNNMEWIILHGFIWLGSC | 15036.5 | 2090 | 4661 | 1147 | 87 | 1210.5 | 180.5 |
| HKVRSMAYHLVFFEEDEGSC | 2063.5 | 1281 | 1016 | 719 | 224.5 | 1246.5 | 199 |
| MHAHNPLYIHLNYLDHPGSC | 9254.5 | 2799.5 | 772.5 | 2175.5 | 101.5 | 2195 | 387.5 |
| WWGREGWEREKRTTWLKGSC | 10785 | 2936 | 3696.5 | 5510 | 494.5 | 4978.5 | 1912.5 |
| EHGQPQPSHDWYGVFRYGSC | 65535 | 65535 | 48343.5 | 65535 | 99.5 | 65535 | 10897 |
| AMYKYHRPIATRMLPLFGSC | 37538.5 | 31418 | 15094.5 | 49915 | 504.5 | 28007.5 | 11693.5 |
| EGNGWSGVNGNLFPRQGGSC | 65535 | 65535 | 60509.5 | 64212 | 388 | 65535 | 9449 |
| ESAHSLWFGWRSVRHFDGSC | 42010 | 25381.5 | 20082.5 | 41735 | 215 | 38024.5 | 3509 |
| QFSKGQTIIFVPQKFKEGSC | 65535 | 57188 | 31536 | 32231 | 226.5 | 21730 | 2918 |
| EGWHALLQFARDNWKPWGSC | 65535 | 65535 | 64089 | 65535 | 300 | 60838 | 6683 |
