# Supplementary material for: A General Method to Discover Epitopes from Sera
Source: PLoS One. 2016 Jun 14;11(6):e0157462. doi: 10.1371/journal.pone.0157462 (PMC4907474; doi:10.1371/journal.pone.0157462)
Supplement: S4 Table — (DOCX) [file pone.0157462.s004.docx]

**Supplementary Table 4**. GLAM2 analysis of the sequence similarity between 108 selected array peptides and the SMCfs peptide.

| **Peptide** | **Glam2Score** | **Matching SMC1fs Peptide Sequence** | **Matching Peptide Sequence** |
| --- | --- | --- | --- |
| RWIYTHHLADRVRRKGP | 8.6979 | IYCH | IYTH |
| WGIYASWKHDNPGSMMY | 8.52623 | GIY | GIY |
| ERWDESQGMWWQVEPQW | 8.50097 | GIYCHEEPQ | GMWWQVEPQ |
| TISKYVMVEPMRQHEEW | 7.85694 | HEE | HEE |
| EHGQPQPSHDWYGVFRY | 7.52681 | HEEPQ | HGQPQ |
| MHAHNPLYIHLNYLDHP | 7.3654 | IYCH | LYIH |
| RNHDESSRNKNHYKNDY | 7.23444 | HEEPQR | HDESSR |
| RVGEMPMREYDISGGSG | 7.12031 | EEPQRE | EMPMRE |
| KSHDTNEESSNRQDSNK | 6.96457 | EEPQREDS | ESSNRQDS |
| ALGLMLALYSHGGKWPD | 6.7684 | IYCH | LYSH |
| VWGKGGMYEAHYRRNGE | 6.51185 | GIY | GMY |
| LRKISRGIWGMREAGEF | 6.40025 | GIY | GIW |
| RMHPRLSAFQWNNDNSI | 6.39226 | DSSI | DNSI |
| FPRKRNWWNTGPMREMN | 6.16974 | PQRE | PMRE |
| AVSHQEMNEGEQGPMRE | 6.16974 | PQRE | PMRE |
| LGGLSPMRETVVWWHWH | 6.16974 | PQRE | PMRE |
| VGPYDNQNYTIWRYTHF | 6.13596 | YCH | YTH |
| GGEKRRKNATKHEQWIL | 5.95133 | HEE | HEQ |
| PTYHIALIDELGAQYSH | 5.9222 | YCH | YSH |
| EPKLWFKPRRGGYRHRH | 5.92144 | GIYCH | GGYRH |
| KMNGQGMKYWHWSRAQY | 5.84463 | YCH | YWH |
| TAFYRTLTKHEVDPGIA | 5.78213 | HE | HE |
| PMHEVIQWYTQADMHAD | 5.78213 | HE | HE |
| KHEMWNWVFLTVNKERV | 5.78213 | HE | HE |
| KHTAFHNHETVRVHSWF | 5.78213 | HE | HE |
| DTGDMNPGYNHIWRTRN | 5.54059 | YCH | YNH |
| LPHYPYQFMPWFSGWYW | 5.49945 | GIY | GWY |
| SKPKRVMRNWNSQSWDP | 5.32199 | EPQR | KPKR |
| QFSKGQTIIFVPQKFKE | 5.2554 | PQR | PQK |
| FVYRRGIVPTVGKVKRQ | 5.2 | GI | GI |
| MIGMTRHHGIVMPFGSH | 5.2 | GI | GI |
| TGILKPKDDPMLWSWVM | 5.2 | GI | GI |
| KLNGWTIPAHIEMHFHV | 5.16227 | IYCHEE | IPAHIE |
| IMLHPPWMLIQHTMWNQ | 5.14429 | IYCH | IMLH |
| QRSWFSGKEPKFQRIWK | 5.08369 | EEPQ | KEPK |
| FWPNNMEWIILHGFIWL | 5.06041 | IYCH | IILH |
| WIIKHKDVAKKGTFAGK | 5.05373 | IYCHEE | IIKHKD |
| INVAGRRKYSIFSKERK | 5.0138 | REDSSI | RRKYSI |
| RHWRPKFRKFRWWRWHH | 4.96801 | HEEPQ | HWRPK |
| YFIEVRWSTVSITIHHK | 4.88898 | IYCH | ITIH |
| AMYKYHRPIATRMLPLF | 4.8543 | IYCHEEP | MYKYHRP |
| RWRIIHGEWMLLKKWGH | 4.83018 | HEE | HGE |
| QTERTESWHGEVPIIDL | 4.83018 | HEE | HGE |
| EDRFFMNDIKDRSMRFT | 4.7348 | ED | ED |
| HKVRSMAYHLVFFEEDE | 4.7348 | ED | ED |
| KQHKRDYDDSTENHSHT | 4.69865 | EDSS | DDST |
| MAPLAKILRERYVAKTP | 4.67562 | YCHEEP | YVAKTP |
| KARWNGRNMTAPVYWRN | 4.65266 | IY | VY |
| HTDFTVYMSFDHPGKGQ | 4.65266 | IY | VY |
| RFTWFGMWAAMFKPRPQ | 4.64517 | PQ | PQ |
| EREIRPNQVWMENIWFM | 4.60005 | QRE | ERE |
| AWNGQTIEREHMLGWPV | 4.60005 | QRE | ERE |
| WWGREGWEREKRTTWLK | 4.60005 | QRE | ERE |
| GMTKHYYQYPDSKKTLK | 4.54714 | QREDS | QYPDS |
| QLHHWMSSDWAGPFQHV | 4.52515 | GIYCH | GPFQH |
| GKIRFMSFMKGWNIHNI | 4.50092 | GIYCH | GWNIH |
| QIGSYNWLVHAPFAKLM | 4.49418 | GIY | GSY |
| ESHDQRTVQLKRQPIHW | 4.48475 | HEE | HDQ |
| MNSGVRWLHSYYKESHM | 4.45062 | YCHE | YYKE |
| GYREILLLHHAQSRKVQ | 4.43085 | RE | RE |
| PGKDRADWKHYGNYYPT | 4.41721 | RED | RAD |
| LVWLMSTMHGGDNQIHD | 4.31555 | HE | HD |
| KSHDLGNDRSMKFRNRG | 4.31555 | HE | HD |
| WKKLYDKFQQRLTHMAD | 4.29818 | QR | QR |
| MHSDVNSIRQRLYKNKM | 4.29818 | QR | QR |
| NGYRINDHTPNQKPYSY | 4.23937 | IYCHEEPQR | INDHTPNQK |
| VFQTYHWVNSNALLYNP | 4.21536 | IY | LY |
| HPTKMHQPHHLYWSLVQ | 4.21536 | IY | LY |
| HRFRFWKRWRKRRWFHK | 4.21045 | YCHE | WFHK |
| ETDSQQNYKYNKRDKRT | 4.0881 | DS | DS |
| YWVDSWPHFADNLTTRL | 4.0881 | DS | DS |
| NHKAVSNHHAYGDYFWS | 4.06771 | GIY | GDY |
| DQMLMMQQQNTRPPRVF | 4.05894 | PQR | PPR |
| WDYADINRYTAQEHTHT | 3.99106 | YCHEE | YTAQE |
| IFRYVKDFAKADTHKWM | 3.94501 | IY | IF |
| AFLWMTNISPTIFYSAR | 3.94501 | IY | IF |
| KDKGVSPGHFHKMTWKF | 3.92155 | GI | GV |
| DGDTVWRLPKSRFVGVI | 3.92155 | GI | GV |
| EGNGWSGVNGNLFPRQG | 3.92155 | GI | GV |
| PMWLKTYHSSWYNSSHK | 3.90913 | HE | HK |
| WWFKKWFKKFRHFPWHK | 3.90913 | HE | HK |
| AEQNIQSSGMHAMRDRD | 3.84745 | HEEPQRE | HAMRDRD |
| IVKYWSFNQFRIHRQWS | 3.75015 | HEE | HRQ |
| NDAGTIVIGHNQYLNGM | 3.68628 | HEE | HNQ |
| MQMPSFYRGSLPDKHST | 3.67739 | IY | FY |
| TYKMVRVGHFYSYVAFR | 3.67739 | IY | FY |
| SGMHIVLRNGKMFEYSM | 3.63625 | GIY | GMH |
| TLNKRRSWRDGFTADEY | 3.60973 | GIYCHE | GFTADE |
| HNVIEVERKGQKMQGQF | 3.57993 | EPQRE | EVERK |
| EGWHALLQFARDNWKPW | 3.50311 | RED | RDN |
| VGLPAIGNRRRKFKRII | 3.48425 | GI | GL |
| WTGLSEGKERGRGRLWL | 3.48425 | GI | GL |
| TNWMKHIIPNVFAFVNN | 3.45403 | HEEP | HIIP |
| WNHMDVDNFHYVETYRY | 3.41461 | YCH | WNH |
| HHMFMMEWMWSALHPGH | 3.38261 | HE | HH |
| QSQYDQSNESESNSYTD | 3.37828 | DSS | DQS |
| ESAHSLWFGWRSVRHFD | 3.33172 | CH | AH |
| KNRWPAATRYHATIKQW | 3.31804 | REDSSI | RYHATI |
| DMTRVESQQTHTPVQIA | 3.30068 | EPQ | ESQ |
| HYNRYMVIIGNWGKQPI | 3.07403 | EEP | KQP |
| HFSKESWKERLVSTAVG | 3.02055 | EEPQRE | KESWKE |
| EYSMRFKWKWMKPGSFR | 2.90483 | EP | KP |
| NPAWQAMTDILIGYNRP | 2.83704 | HEEP | YNRP |
| FIQTGNRRRVFQWGTNG | 2.66657 | IY | VF |
| WRWWFKRWRFRRKWHWF | 2.55785 | RE | RK |
| RSALTGKGRLAEKTEKA | 2.31955 | EE | EK |
| VLAIILIIVLIAIVLII | 2.3095 | SI | AI |
